# Aquaporin-4 Immunoglobulin G–seropositive Neuromyelitis Optica Spectrum Disorder MRI Characteristics: Data Analysis from the International Real-World PAMRINO Study Cohort

**DOI:** 10.1148/radiol.233099

**Published:** 2024-11-12

**Authors:** Claudia Chien, Vera Cruz e Silva, Emanuel Geiter, Dominik Meier, Hanna Zimmermann, Denis B. Bichuetti, Marcos I. Idagawa, Ayse Altintas, Uygur Tanriverdi, Sasitorn Siritho, Lehka Pandit, Anitha Dcunha, Maria J. Sá, Rita Figueiredo, Peiqing Qian, Caryl Tongco, Itay Lotan, Vadim Khasminsky, Mark A. Hellmann, Hadas Stiebel-Kalish, Dalia L. Rotstein, Lindsay Waxman, Daniel Ontaneda, Kunio Nakamura, Hesham Abboud, M. Omar Subei, Yang Mao-Draayer, Joachim Havla, Nasrin Asgari, Pernille B. Skejø, Ilya Kister, Marius Ringelstein, Simon Broadley, Simon Arnett, Brie Marron, Anna M. Jolley, Michael Wunderlich, Sean Green, Lawrence J. Cook, Michael R. Yeaman, Terry J. Smith, Alexander U. Brandt, Jens Wuerfel, Friedemann Paul, for the Guthy-Jackson Charitable Foundation International Clinical Consortium for NMOSD

**Affiliations:** From the Experimental and Clinical Research Center, Charité-Universitätsmedizin Berlin, corporate member of Freie Universität Berlin, Humboldt-Universität zu Berlin & Max Delbrück Center for Molecular Medicine in the Helmholtz Association, Lindenberger Weg 80, 13125 Berlin, Germany (C.C., H.Z., A.U.B., F.P.); NeuroCure Clinical Research Ctr (C.C., H.Z., A.U.B., J.W., F.P.), Dept of Psychiatry and Neurosciences, Charité-Universitätsmedizin Berlin, Freie Universität Berlin, Humboldt-Universität zu Berlin, Berlin, Germany (C.C.); Medical Image Analysis Center, Basel, Switzerland (V.C.e.S., E.G., D.M.); Paulista School of Medicine, Dept of Neurology and Neurosurgery (D.B.B.), Dept of Diagnostic Imaging, Universidade Federal de São Paulo, São Paulo, Brazil (M.I.I.); Koc Univ, School of Medicine Neurology Dept and Istanbul Univ, Cerrahpasa School of Medicine, Neurology Dept, Istanbul, Turkey (A.A.); Dept of Neurology, Istanbul Univ, Cerrahpasa Faculty of Medicine, Istanbul, Turkey (U.T.); Div of Neurology, Dept of Medicine, Siriraj Hosp, Mahidol Univ, Bangkok, Thailand (S.S.); Bumrungrad International Hosp, Bangkok, Thailand (S.S.); Center for Advanced Neurologic Research, KS Hegde Medical Academy, Nitte Univ, Mangalore, India (L.P., A.D.); Dept of Neurology, Hosp de S. João, Al. Hernâni Monteiro, Porto, Portugal (M.J.S., R.F.); MS Center at Swedish Neuroscience Inst, Seattle, Wash (P.Q., C.T.); Dept of Neurology and Neuroimmunology Clinic, Rabin Medical Center, Petach Tikva, Israel (I.L.); Sackler Faculty of Medicine & Felsenstein Medical Research Center, Tel Aviv Univ, Tel Aviv, Israel (I.L., H.S.K.); Dept of Radiology, Rabin Medical Center, Beilinson Hosp, Israel, and Sackler Faculty of Medicine, Tel-Aviv Univ, Tel Aviv, Israel (V.K.); Dept of Neurology and Neuroimmunology, Rabin Medical Center, Beilinson Hosp, Israel, and Sackler Faculty of Medicine, Tel-Aviv Univ, Tel Aviv, Israel (M.A.H.); Neuro-Ophthalmology Div, Dept of Ophthalmology, Rabin Medical Center, Petah Tikva, Israel (H.S.K.); Div of Neurology, Univ of Toronto, St Michael’s Hosp, Toronto, Canada (D.L.R., L.W.); Mellen Center, Cleveland Clinic, Cleveland, Ohio (D.O.), Dept of Biomedical Engineering, Cleveland Clinic, Cleveland, Ohio (K.N.); Multiple Sclerosis and Neuroimmunology Program, Univ Hosps of Cleveland, Case Western Reserve Univ School of Medicine, Cleveland, Ohio (H.A., M.O.S.); Michigan Inst for Neurologic Disorders, Farmington Hills, Mich (Y.M.D.); Inst of Clinical Neuroimmunology, LMU Hosp, Ludwig-Maximillians Universität München, Munich, Germany (J.H.); Dept of Neurology, Slagelse Hosps, Odense, Denmark (N.A.); Insts of Regional Health Research & Molecular Medicine, Univ of Southern Denmark, Odense, Denmark (N.A.); Dept of Radiology, Aleris Hosp, Copenhagen, Denmark (P.B.S.); NYU Multiple Sclerosis Comprehensive Care Center, Dept of Neurology, NYU School of Medicine, New York, NY (I.K.); Dept of Neurology, Center for Neurology and Neuropsychiatry, LVR-Klinikum, Heinrich Heine Univ Düsseldorf, Düsseldorf, Germany (M.R.); School of Medicine and Dentistry, Gold Coast Campus, Griffith Univ, Queensland, Australia (S.B., S.A.); Dept of Neurology, Gold Coast Univ Hosp, Queensland, Australia (S.A.); Dept of Pediatrics, Univ of Utah, Salt Lake City, Utah (B.M., A.M.J., M.W., S.G., L.J.C.); Dept of Medicine, Divs of Molecular Medicine & Infectious Diseases, and Ludquist Inst for Biomedical Innovation at Harbor-UCLA Medical Center, Torrance, Calif (M.R.Y.); Dept of Medicine, David Geffen School of Medicine at UCLA, Los Angeles, Calif (M.R.Y.); Depts of Ophthalmology and Visual Sciences, Kellogg Eye Center, Univ of Michigan, Ann Arbor, Mich (T.J.S.); Div of Metabolism, Endocrine and Diabetes, Dept of Internal Medicine, Univ of Michigan Medical School, Ann Arbor, Mich (T.J.S.); Hoffmann-LaRoche, Basel, Switzerland (J.W.); Dept of Neurology, Charité-Universitätsmedizin Berlin, Germany (F.P.); Affiliated author members of the Guthy-Jackson Charitable Foundation (GJCF) International Clinical Consortium (ICC) for NMOSD are listed in Appendix S1.

## Abstract

**Background:**

Patients with neuromyelitis optica spectrum disorder (NMOSD) are often seropositive for antibodies against aquaporin-4 (AQP4). The importance of MRI monitoring in this disease requires evaluation.

**Purpose:**

To profile MRI features from a large international cohort with AQP4 immunoglobulin G (IgG)-seropositive NMOSD (from the Parallel MRI in NMOSD [PAMRINO] study) and to evaluate and confirm existing knowledge regarding the incidence, location, and longitudinal development of characteristic lesions in the central nervous system associated with AQP4-IgG–seropositive NMOSD.

**Materials and Methods:**

In this retrospective study (from August 2016 to January 2019), MRI and clinical data were collected from 17 NMOSD expert sites in 11 countries across four continents. Clinical features and lesions identified at cross-sectional and longitudinal MRI were assessed. No formal statistical tests were used to compare observations; however, means, SDs, and 95% CIs are reported when evaluating lesion frequencies.

**Results:**

Available T1-weighted and T2-weighted MRI scans in patients with AQP4-IgG–seropositive NMOSD (*n* = 525) were read. Among the 525 patients, 320 underwent cerebral MRI examinations with T2-weighted hyperintense cerebral (264 of 320; 82.5%), cerebellar (44 of 320; 13.8%), and brainstem (158 of 321 [49.2%], including one lesion observed at cervical spinal cord [SC] MRI) lesions. Lesions in the optic nerves, analyzed from 152 MRI examinations, were mainly found in the central (81 of 92; 88%) and posterior (79 of 92; 86%) sections (bilaterally in 39 of 92; 42%). Longitudinally extensive transverse myelitis was the predominant SC lesion pattern (upper compartment from 322 MRI examinations, 133 of 210 [63.3%]; and lower compartment from 301 MRI examinations, 149 of 212 [70.3%]). However, nonlongitudinal extensive transverse myelitis lesions were also observed frequently (105 of 210; 50.0%) in the cervical SC. Clinical data (*n* = 349; mean age, 44 years ± 14 [SD]; 202 female patients) and acute lesions at contrast-enhanced (CE) MRI (*n* = 58, performed within 30 days of the last attack) were evaluated. CE lesions were detected in the cerebrum (eight of 13; 62%), optic nerves (14 of 19; 74%), or chiasm (three of four; 75%) within 15 days of any relapse. In the upper SC (29 of 44; 66%), CE lesions were frequently observed up to 20 days after a clinical myelitis event.

**Conclusion:**

A high incidence of abnormal brain MRI examinations and nonlongitudinal extensive SC lesions was found in patients in PAMRINO with AQP4-IgG–seropositive NMOSD.

© The Author(s) 2024. Published by the Radiological Society of North America under a CC BY 4.0 license.

*Supplemental material is available for this article.*

## Graphical abstract

**Figure:**
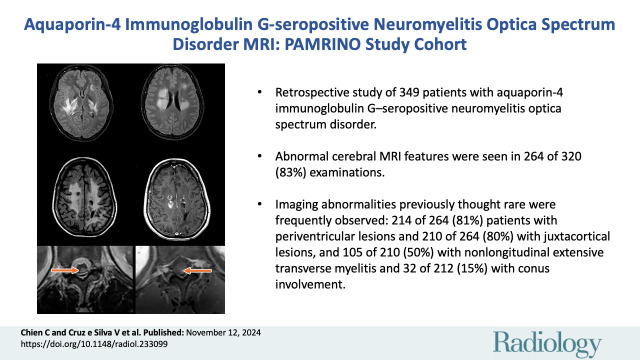

**Summary**

Large, international, real-world MRI assessments showed high heterogeneity in the data collected from patients with aquaporin-4 immunoglobulin G–seropositive neuromyelitis optica spectrum disorder and frequent cerebral and lower spinal cord abnormalities.

**Key Results**

■ In this retrospective study of 349 patients with aquaporin-4 immunoglobulin G–seropositive neuromyelitis optica spectrum disorder, abnormal cerebral MRI features were found in 264 of 320 (82.5%) of brain MRI examinations.
■ Imaging abnormalities previously found in a minority of patients were frequently observed: 214 of 264 (81.1%) patients with periventricular-lateral ventricles and 210 of 264 (79.5%) with juxtacortical lesions, and 105 of 210 (50%) with nonlongitudinal extensive transverse myelitis and 32 of 212 with conus involvement (15.1%).
■ Contrast enhancement could be visualized in 14 of 19 (74%) optic nerve lesions up to 15 days after acute optic neuritis; and in 29 of 44 (66%) upper spinal cord lesions up to 20 days after acute myelitis attack.

## Introduction

Neuromyelitis optica spectrum disorder (NMOSD) is an autoimmune inflammatory central nervous system disease characterized by a severe onset and relapse events, including optic neuritis, longitudinally extensive transverse myelitis (LETM), brainstem and/or hypothalamic syndromes, and encephalitis (1). Serum immunoglobulin G (IgG) antibodies against the astrocytic water channel aquaporin-4 (AQP4) are found in most patients (2). Brain parenchymal lesions that appear hyperintense at T2-weighted MRI occur predominantly in areas with high AQP4 protein expression, such as in the area postrema or the periependymal tissue adjacent to the third, fourth, or lateral ventricles (3). Spinal cord (SC) lesions have also been described, not all of which involve LETM (4,5). To date, published MRI analyses mostly originate from relatively small and/or monocentric studies. Because of the rarity of the disease, as well as the lack of internationally standardized imaging protocols, information is scarce regarding the regional lesion distribution, frequency, and acute attack–related MRI enhancement. Therefore, the importance of MRI in monitoring the disease course requires further evaluation (6).

The Parallel MRI in NMOSD (PAMRINO) study enables large-scale radiologic readings in patients from different sites, and with different clinical presentations. The aim of this study was to descriptively profile MRI features from this real-world, large, international cohort with AQP4-IgG–seropositive NMOSD, and to evaluate and confirm existing knowledge regarding the incidence, location, and longitudinal development of characteristic central nervous system lesions associated with AQP4-IgG–seropositive NMOSD.

## Materials and Methods

F. Hoffmann-La Roche provided financial support in the form of an independent grant to the coordination and analysis centers. At the time of the research and study drafting, none of the authors were employees of Roche. The researchers retained complete control over the design, methods, and conduct of the research, data, and information submitted for publication. The funding did not influence the results or conclusions of the study. Please see also Appendix S1 (supplemental methods section) for additional information regarding funding for this study.

### Study Design and Participants

The institutional review boards of each center approved this retrospective study; written informed consent was obtained from all participants. Expert centers affiliated with the Guthy-Jackson Charitable Foundation International Clinical Consortium network were recruited to participate between August 2016 and January 2019. For this study, patients in the PAMRINO study with AQP4-IgG–seropositive NMOSD (2015 International Panel criteria [7]) were selected exclusively.

### Meta-analysis

A literature search on the clinical prevalence and MRI findings in population- and hospital-based cohorts of patients with NMOSD who were seropositive and seronegative for AQP4-IgG was performed. PubMed was searched for articles published in English from database inception to February 6, 2024, with the following search terms: [prevalence] OR [MRI] OR [multicenter] AND [NMOSD] (Table S1). Studies regarding adults with NMOSD with clinical attack and MRI information were included (*n* = 32). The pooled mean prevalences, SDs, and IQRs were calculated for each clinical and MRI parameter (Table S2) using software (R, version 4.3.0, R Project for Statistical Computing; R meta package, version 7.0–0, *https://github.com/guido-s/meta/*), where the inverse variance method for pooling was used. Exclusion criteria are detailed in Appendix S1 (supplemental methods section).

### Data Collection

Anonymized demographic and clinical disability data were entered into standardized electronic clinical record forms in Research Electronic Data Capture, or REDCap (Vanderbilt, *https://projectredcap.org/software/*) (8), by participating centers (Appendix S1, supplemental methods section). Pseudonymized Digital Imaging and Communications in Medicine data were collected without restrictions for magnetic field strength, scanner manufacturer, or type of sequence. Clinical visit data were not restricted to coincide with MRI visit dates because this is a rare disease (9), and many expert centers previously had no standardized MRI or clinical visit protocols for diagnosing and/or evaluating these patients until the past 5 years. Further data collection details are found in Appendix S1 (supplemental methods section).

### Data Cleaning, MRI, and Clinical Data Matching

Technical MRI data were extracted from Digital Imaging and Communications in Medicine headers. MRI sequence data collected as a part of the clinical routine at participating sites are detailed in Appendix S1 (supplemental methods section) and Tables S3–S6. PAMRINO clinical data from REDCap were exported in full and filtered to include only patients with AQP4-IgG–seropositive NMOSD. Patients tested using cell-based or immunofluorescence antibody assays positive for AQP4-IgG at any time during their clinical history were considered to have AQP4-IgG–seropositive NMOSD (10). Patients with MRI examinations, even without matched clinical visit data, were included in this study for further cross-sectional (*n* = 349) and longitudinal radiologic readings (*n* = 220).

### MRI Lesion Analyses

MRI scans were independently read by a board-certified neuroradiologist (V.C.e.S., with >12 years of experience). Further radiologic reading quality check information can be found in Appendix S1 (supplemental methods section). Readings were performed blinded to patient identity and clinical or laboratory information. Imaging features were visually assessed and classified using available T2-weighted and unenhanced and/or contrast-enhanced (CE) T1-weighted sequences, and lesions were sorted according to their anatomic location. The following compartments were rated: brain parenchyma, cerebellum, and brainstem; optic nerve (ON) and optic chiasm; and SC–upper SC (the cervical cord) and lower SC (the thoracic cord and conus medullaris).

In addition to characteristic NMOSD lesions (7), brain lesions resembling small-vessel disease were investigated (11). ON lesions were assessed in anterior, central, and posterior (excluding the chiasm) sections according to the intraorbital, intracanalicular, and intracranial anatomic segments (12). Acute ON and chiasmatic lesions were defined as T2-weighted hyperintense lesions with a tumefactive effect, and concomitant CE images were assessed, when available, to help confirm acute inflammation. SC lesions extending over three or more vertebrae were considered LETM (13). Nonlongitudinal extensive transverse myelitis (NETM) was defined as a lesion length of fewer than three vertebrae (14). SC lesion topography was recorded according to the lateral, central, posterior or anterior, and ventral region or regions affected. So-called bright spotty lesions (15) were evaluated in the SC. SC atrophy was visually rated as general or focal (16). For follow-up MRI, a minimum 1-month interval between visits was established to define relapsing disease according to the International Consensus Diagnosis Criteria (7).

### Clinical and MRI Phenotyping

**Figure 1:**
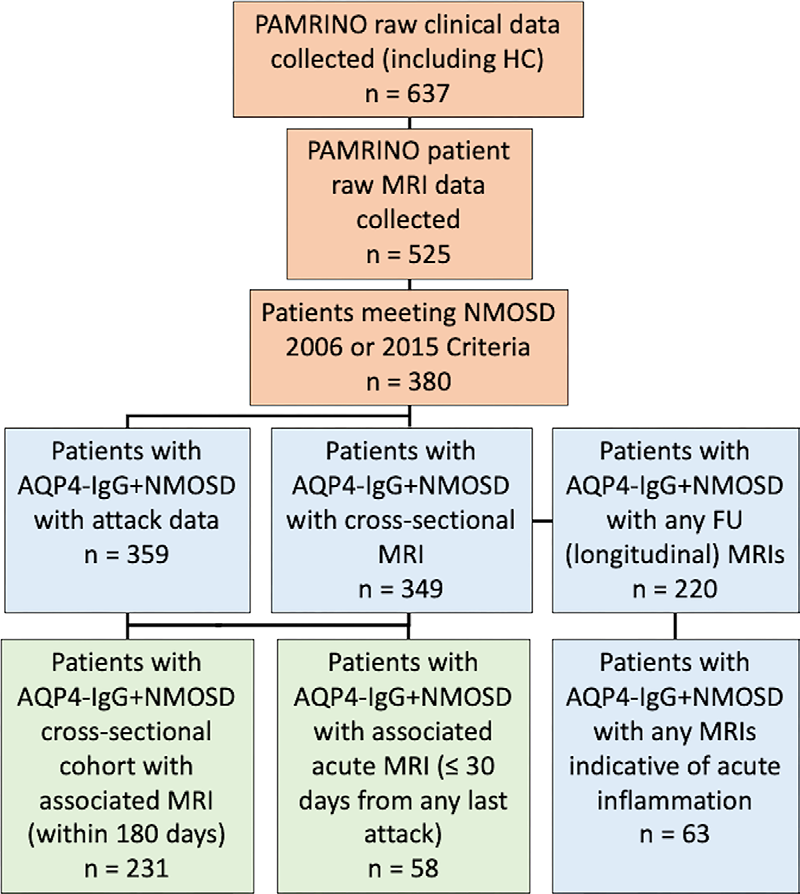
Flowchart shows an overview of the data collection, quality checks, cleaning, and matching performed as part of the Parallel MRI in NMOSD (PAMRINO) study. Figure shows data collection and cleaning steps (orange), data used for separate clinical phenotyping and MRI radiologic reading sections (blue), and data used in the matched MRI data demographics and acute MRI analysis sections of this study (green). No MRI scans in patients with aquaporin-4 (AQP4) immunoglobulin G–seropositive (IgG+) neuromyelitis optica spectrum disorder (NMOSD) were excluded from reading. FU = follow-up, HC = healthy control.

MRI scans were matched to each clinical visit based on the examination date (within the previous 180 days or within 180 days after the MRI examination date). The first-matched MRI scans (Fig 1) per patient (hereafter, referred to as baseline) were chosen as the cross-sectional cohort for phenotyping. Baseline AQP4-IgG–seropositive NMOSD data (*n* = 231) were analyzed for demographic features, attack types, and general disability as measured by the Expanded Disability Status Scale (Table 1). The acute MRI and clinical phenotyping methods used are described in Appendix S1 (supplemental methods section).

**Table 1:**
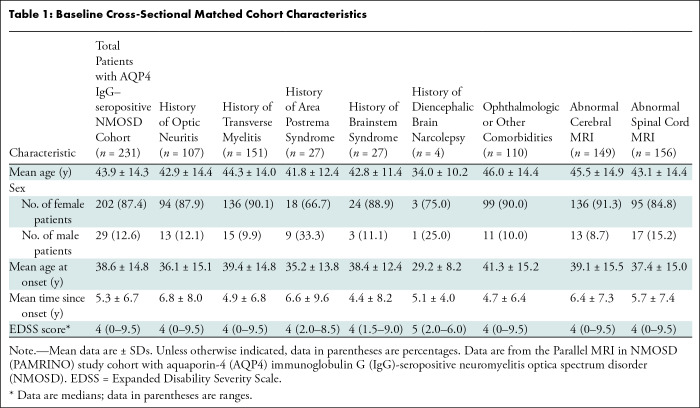
Baseline Cross-Sectional Matched Cohort Characteristics

### MRI Analysis of Acute Inflammation and Summary of Findings

If CE MRI examinations of acute inflammation were available, the duration of CE after a disease-related attack was visualized graphically in different compartments.

### Statistical Analysis

This is a descriptive study. Referenced values specified in this study indicate the number of patients; however, individual MRI findings may differ in total number of patients according to availability.

No formal statistical tests were used to compare observations; however, means, SDs, and 95% CIs are reported when evaluating lesion frequencies. The graphs were produced using software (R, version 4.3.0; R Project for Statistical Computing).

## Results

### Baseline Demographics

A total of 891 MRI examinations in 349 patients with AQP4-IgG–seropositive NMOSD were evaluated. The number of patients with available MRI examinations in each compartment are described. Figure 1 illustrates the data-cleaning procedure, with patients included in this study for further cross-sectional (*n* = 349) and longitudinal radiologic reading (*n* = 220). Matched clinical and MRI data in 231 patients with AQP4-IgG–seropositive NMOSD were included in this study (mean age, 43.9 years ± 14.3 [SD]; 202 female patients, 29 male patients). Table 1 lists all relevant patient demographic features from the baseline cross-sectional matched cohort.

MRI scans acquired within 30 days of relapse (referred to as acute MRI scans) were analyzed (158 scans in 63 patients) (Fig 1).

### Cross-Sectional MRI Overview

MRI scans from patients with AQP4-IgG–seropositive NMOSD were analyzed (Fig 1), regardless of matching clinical information (Table 2). Because patients may not have undergone MRI in every anatomic compartment and may have simultaneous lesions in different central nervous system regions, the total number of patients per region is not equal, as shown in Table 2.

**Table 2:**
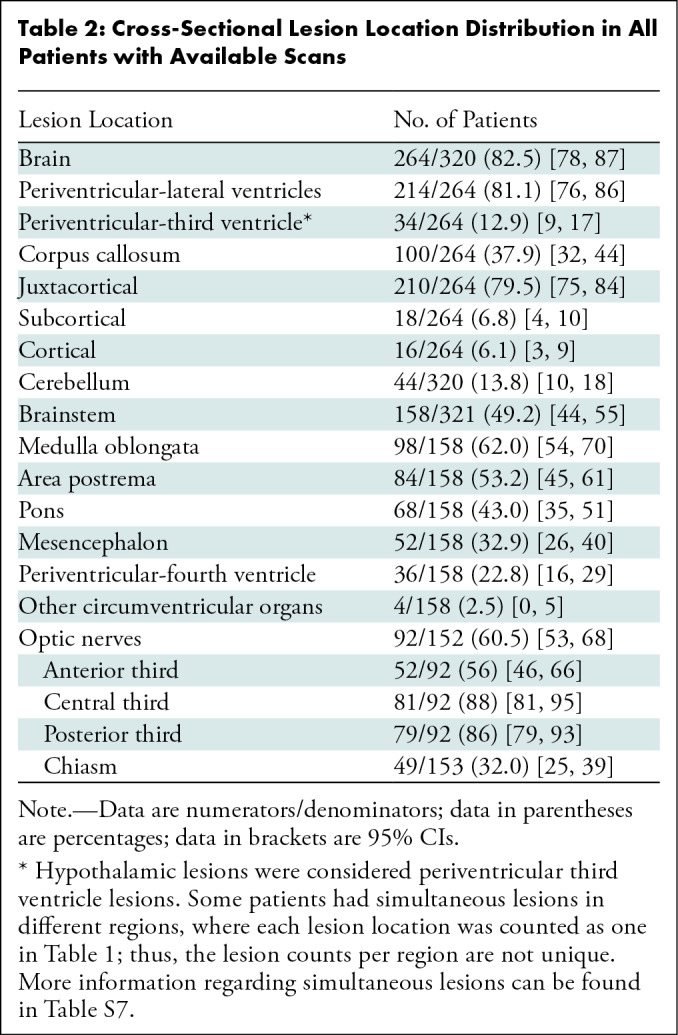
Cross-Sectional Lesion Location Distribution in All Patients with Available Scans

### Cross-Sectional MRI Findings

***Brain.—***Brain MRI scans were available for lesion evaluation in 320 of 349 patients (Table S4). Cerebral imaging showed abnormalities in 264 of 320 (Table 2) of the patients from PAMRINO with AQP4-IgG–seropositive NMOSD (82.5%, compared with the mean pooled average of 46.2% from the literature meta-analysis).

**Figure 2:**
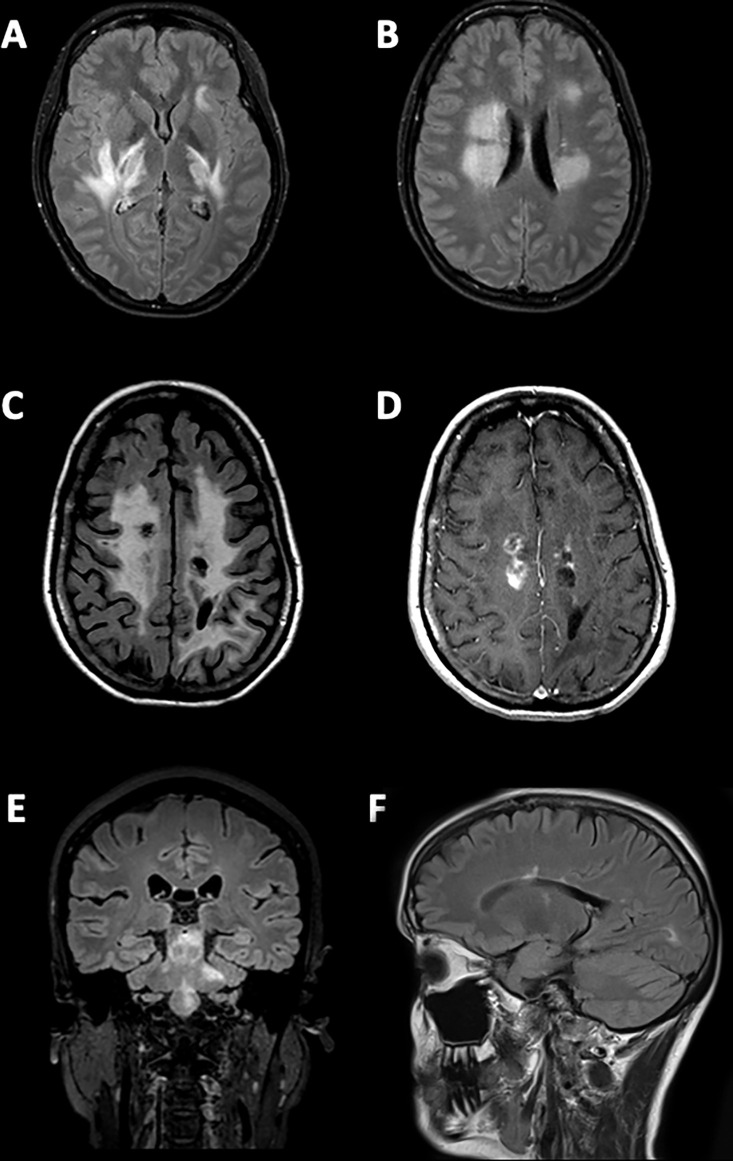
Representative scans from patients with aquaporin-4 immunoglobulin G–seropositive neuromyelitis optica spectrum disorder **(A, B)** Axial T2-weighted fluid-attenuated inversion recovery MRI scans show a 20-year-old female patient with a previous symptomatic narcolepsy or acute diencephalic clinical syndrome. Images show **(A)** bilateral tumefactive lesions extending throughout the internal capsules and external capsules and involving both thalami, with a left subcortical frontal-opercular lesion and **(B)** bilateral fluffy lesions of the deep and periventricular white matter, with ependymal involvement. **(C)** A non–fat-saturated axial T2-weighted fluid-attenuated inversion recovery image and **(D)** a contrast-enhanced T1-weighted MRI scan in a 19-year-old female patient with previous optic neuritis show bilateral extensive abnormalities in the white matter, nontumefactive, with vacuolization and concomitant contrast enhancement. **(E)** A coronal T2-weighted fluid-attenuated inversion recovery MRI scan in a 55-year-old female patient with previous optic neuritis and brainstem involvement shows extensive brainstem involvement with middle cerebellar peduncles. **(F)** A sagittal T2-weighted fluid-attenuated inversion recovery MRI scan in a 38-year-old female patient with previous myelitis shows a Dawson finger–like lesion.

The hyperintense lesion distributions on T2-weighted or fluid-attenuated inversion recovery images were mostly periventricular (alongside lateral ventricles), with ependymal lining involvement, and in the subcortical regions (Table 2). Tumefactive lesions were observed in 17 of 264 patients (6.4%; Fig 2); the so-called Dawson finger morphologic appearance was identified in 38 of 264 (14.4%) patients; and 50 of 264 patients (18.9%) presented with small multifocal and nonspecific white matter lesions, which resemble small-vessel disease at T2-weighted fluid-attenuated inversion recovery MRI. Lesions in the brain were identified on CE T1-weighted MRI scans in 42 of 245 (17.1%) patients. A central vein sign (17) could be identified in only two of 152 (1.3%) patients with T2*-weighted or susceptibility-weighted imaging sequences.

Brainstem involvement (<50% of patients; Fig 2E) was mostly located in the medulla oblongata and area postrema and less so in the pons or mesencephalon; CE lesions were identified on 41 of 249 (16.5%) lesions, from 249 of 320 (77.8%) available CE MRI scans.

***Optic nerves.—***Among the 349 patients, 152 underwent MRI with an orbital acquisition protocol that could be used to evaluate the ON (Table S4). ON lesions were frequent (>50%), mainly in the central and posterior regions (Table 2). Bilateral ON lesions were identified in 39 of 92 (42%) patients. However, bilateral hyperintense lesions with slight tumefactive effects, characteristic of acute lesions, were observed in only six of 39 (15%) patients. These lesions were characterized by longitudinally extensive ON lesions encompassing more than half of the length of the ON. CE lesions were found in 47 of 92 (51%) patients with available data from CE MRI.

***Optic chiasm.—***MRI was used for distinct evaluation of the optic chiasm in 153 of 349 patients (Table S4). Chiasmatic lesions were observed in fewer than half of the available scans (Table 2), and 24% (12 of 49) of the T2-weighted scans in patient lesions exhibited CE. Lesions exclusively affecting the chiasm were found in five of 49 (10%) patients, whereas simultaneous effects of both the chiasm and ON were identified in 44 of 152 (28.9%) patients, almost always with posterior involvement (43 of 44; 98%). In these patients, bilateral (28 of 44; 64%) rather than unilateral (16 of 44; 36%) ON involvement was observed. However, bilateral tumefactive ON lesions extending to the chiasm, suggestive of acute inflammation, were found in only four of 27 (15%) patients and involved all three sections of the ON.

***SC lesions.—***In 322 of 349 patients with upper SC MRI and 301 of 349 patients with lower SC MRI, SC lesions were evaluated (Table S4). SC lesions (Table 3) were frequently observed (upper SC, 65.2%; lower SC, 70.4%) and were distributed along the entire cord. LETM was the predominant lesion pattern, and CE LETM was observed more often than CE NETM. However, NETM represented up to 50% of the total SC lesions—mostly central—and were isolated in the upper SC in 105 of 210 (50.0%) patients and in the lower SC in 84 of 212 (39.6%) patients. Simultaneous LETM and NETM were not frequently found in either compartment. Conus medullaris involvement was identified in 32 of 212 patients (15.1%) with lesions in the lower SC, isolated in 15 of 32 (47%) patients and by extension of lower SC LETM in 17 of 32 (53%) patients. Bright spotty lesions were identified in 83 of 276 (30.1%) patients with SC lesions and were more often observed in LETM (Table 3). A sagittal T2-weighted hyperintense intramedullary spinal line, described in myelin oligodendrocyte glycoprotein antibody–associated disease (18), was present in 34 of 277 (12.3%) patients with lesions involving the upper and lower SC. Of these patients, 32 of 34 (94%) associated with LETM, two of 34 (6%) associated with NETM, and four of 34 (12%) associated with both lesion types simultaneously. The “H” sign on axial SC images (central cord gray-matter T2-weighted hyperintensity) (18,19), suggestive of myelin oligodendrocyte glycoprotein antibody–associated disease, was observed in 11 of 133 (8.3%) patients with LETM, extending throughout the entire SC in four of 11 (36%) patients. Atrophy was more frequently focal than generalized, mostly affecting the lower SC.

**Table 3:**
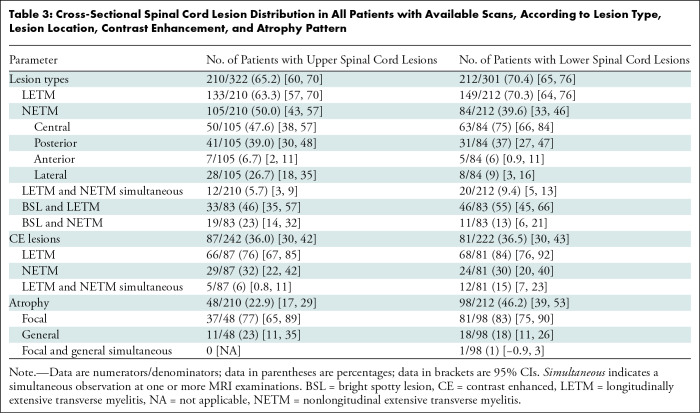
Cross-Sectional Spinal Cord Lesion Distribution in All Patients with Available Scans, According to Lesion Type, Lesion Location, Contrast Enhancement, and Atrophy Pattern

Table S7 details the simultaneous lesions found in different central nervous system compartments that were present in patients with AQP4-IgG–seropositive NMOSD. A high proportion of patients had simultaneous brain and lower SC (126 of 213; 59.2%; 95% CI: 53, 66), brain and upper SC (135 of 247; 54.6%; 95% CI: 48, 61), and brain and ON lesions (73 of 149; 49.0%; 95% CI: 41, 57).

### Longitudinal MRI Data

Follow-up MRI scans were available in 220 patients (compartments are detailed in Table S8). The following radiologic readings included all available longitudinal MRI (*n* = 220; 762 visits). Acute inflammation was found on MRI scans in 63 patients (Fig 1, Table S9).

***Brain MRI.—***Of the 220 patients who had longitudinally acquired MRI scans, 146 had cerebral MRI scans available for analysis (Table S8). Eleven of the 146 (7.5%) patients with initially inconspicuous MRI developed lesions within a follow-up period between 30 days and 6 years after undergoing initial MRI. Complete resolution of brain lesions occurred in four of 146 (2.7%) patients from within 20 days to 2.5 years. However, an increase in brain lesion burden with multifocal distribution was the most frequent pattern observed. New brain lesions frequently occurred subcortically, periventricularly adjacent to the lateral ventricles, and in the corpus callosum (Table S10). For more details regarding CE lesions and brainstem lesions, see Appendix S1 (supplemental results section).

***ON MRI.—***Of the 220 patients with longitudinally acquired MRI, 58 patients had orbital MRI scans available for ON analysis (Table S8). For more details regarding ON atrophy and CE lesions, see Appendix S1 (supplemental results section). Figure S1A shows an example of CE lesions within 24 hours of symptom onset.

***Optic chiasm MRI.—***Among the 220 patients with longitudinally acquired MRI scans, 59 patients had cerebral and/or orbital MRI scans available for chiasm analysis (Table S8). Chronic chiasmatic lesions were identified on follow-up scans in 10 of 59 (17%) patients, whereas new chiasmatic lesions were observed in 12 of 59 (20%) patients, with a maximum of 5 years of follow-up. Previous scans were normal in seven of these 12 patients (58%). For more details regarding chiasmic CE lesions, see Appendix S1 (supplemental results section).

***SC MRI.—***Among the 220 patients with longitudinally acquired MRI scans, 159 patients had upper SC MRI scans and 137 patients had lower SC MRI scans available for analysis (Table S8). The lower SC (106 of 137; 77.4%) and upper SC (18 of 159; 74.2%) were often affected at multiple follow-up visits, with a predominant LETM pattern (72 of 106 [67.9%] in the LSC; 74 of 118 [62.7%] in the upper SC) in both compartments. However, NETM patterns were also observed (72 of 118 [61.0%] in the upper SC; 50 of 106 [47.2%] in the lower SC).

**Figure 3:**
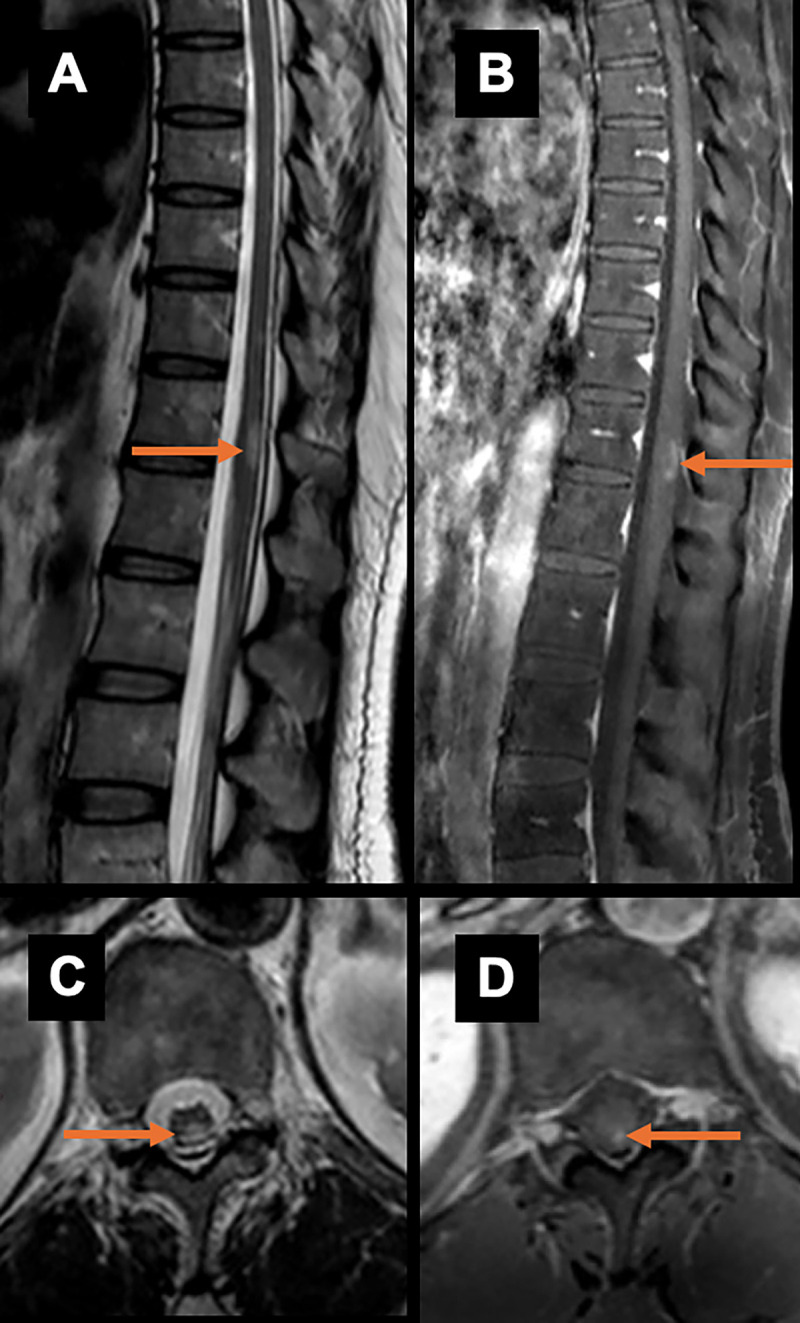
Representative T2 hyperintense **(A, C)** and T1 gadolinium-enhancing **(B, D)** nonlongitudinal extensive transverse myelitis isolated in the conus medullaris from a 22-year-old female patient (arrows).

We also observed isolated involvement of the conus medullaris in patients with AQP4-IgG–seropositive NMOSD (Fig 3), which was previously hypothesized to be more characteristic of myelin oligodendrocyte glycoprotein antibody–associated disease (19). This finding is similar to that of a previous study in which extensions of LETM from the thoracic cord into the conus were described in more than 20% of patients with AQP4-IgG–seropositive NMOSD (20). For more details on new SC lesions and CE lesions, see Appendix S1 (supplemental results section, Table S10) and Figure S1B.

***Clinical and MRI phenotyping.—***Cross-sectional MRI examinations and clinical data in patients from each center with AQP4-IgG–seropositive NMOSD were evaluated for mean differences in age at onset and Expanded Disability Status Scale scores. In this international study, the age at first clinical visit varied between the specialized centers (within less than or more than 10 years from median patient age of approximately 45 years) (Fig S2). Expanded Disability Status Scale scores at the first clinical visit are less variable; however, it should be noted that only 152 of 231 patients (65.8%) had Expanded Disability Status Scale scores collected at the first available visit.

***MRI analysis of acute inflammation and summary of findings.—***To determine the duration of blood-brain barrier breakdown during relapse, data regarding lesion counts from CE T1-weighted MRI and lesion locations within 30 days after relapse were evaluated.

**Figure 4:**
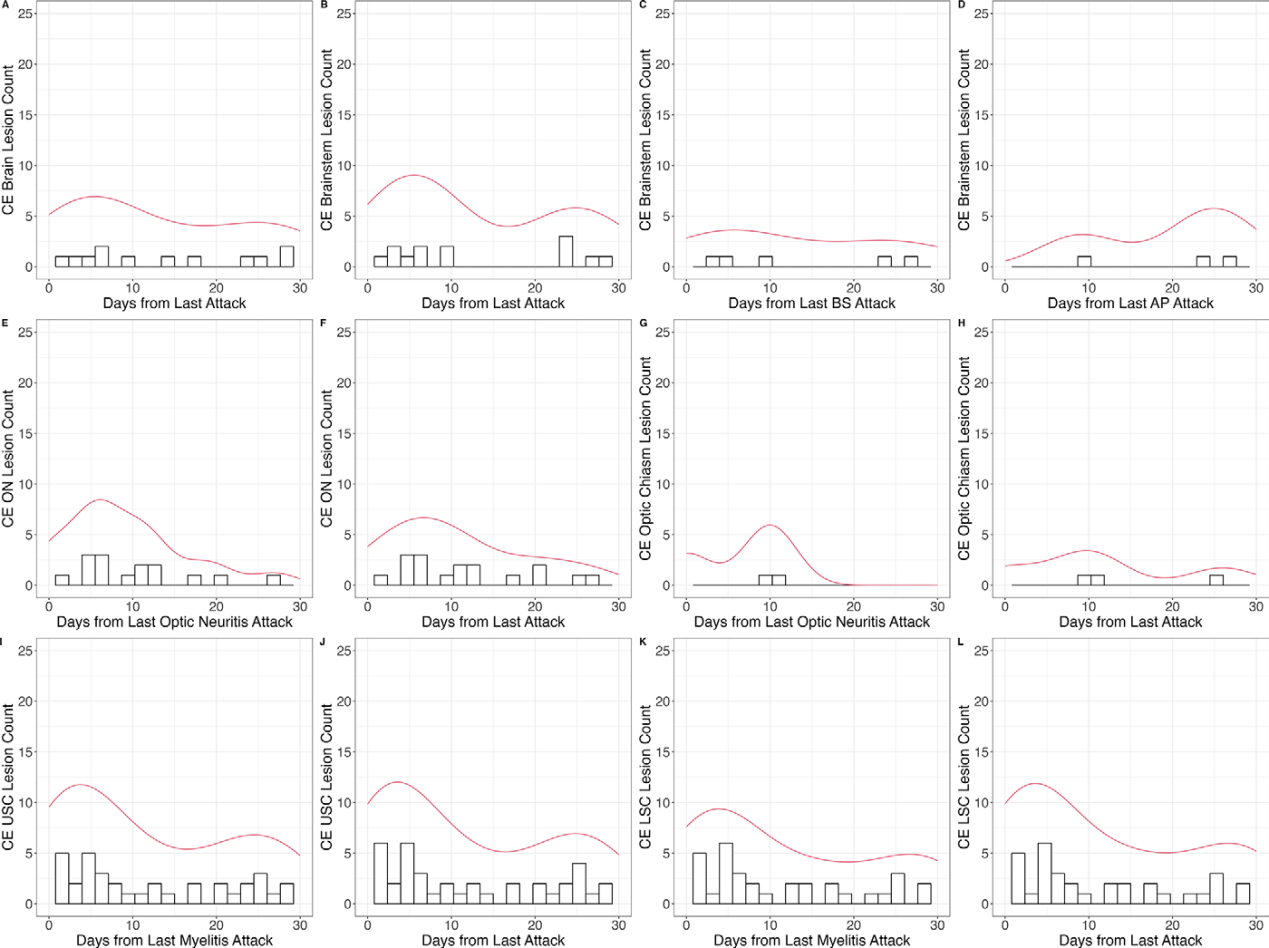
Evaluation of contrast-enhancing lesions in acute relapse MRI scans observed in **(A–D)** the brain and brainstem, **(E–H)** the optic nerve (ON) and optic chiasm, and **(I–L)** the upper spinal cord (USC) and lower spinal cord (LSC). Contrast-enhanced lesions were detected in the cerebrum (eight of 13), optic nerves (14 of 19), or chiasm (three of four) within 15 days of any type of attack. In the upper spinal cord (29 of 44), contrast-enhanced lesions were frequently observed up to 20 days after a clinical myelitis event. The curves indicate density. AP = area postrema, BS = brainstem, CE = contrast enhanced.

In 58 patients, at least one CE MRI scan (101 MRI scans in total from multiple examinations) was available within the acute relapse setting. The frequency of observed CE in lesions in the upper SC and lower SC slightly increased 20 days after myelitis relapse, resulting in bimodal distributions (Fig 4).

**Figure 5:**
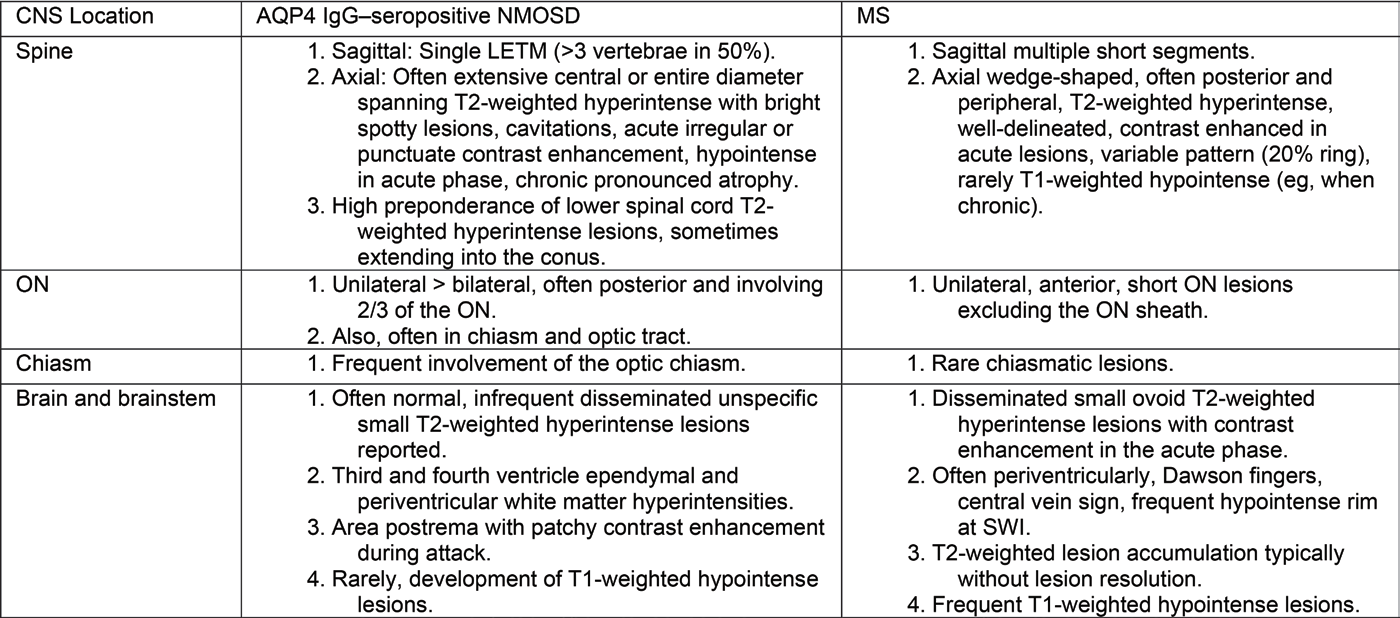
Overview of characteristic MRI features differentiating between aquaporin-4 (AQP4) immunoglobulin G (IgG)-seropositive neuromyelitis optica spectrum disorder (NMOSD) and multiple sclerosis (MS). CNS = central nervous system, LETM = longitudinally extensive transverse myelitis, ON = optic nerve, SWI = susceptibility-weighted imaging.

For more information regarding CE lesions, see Appendix S1 (supplemental results section). There are several MRI indications that are more commonly associated with AQP4-IgG–seropositive NMOSD MRI than multiple sclerosis in clinical practice (Fig 5).

**Figure 6:**
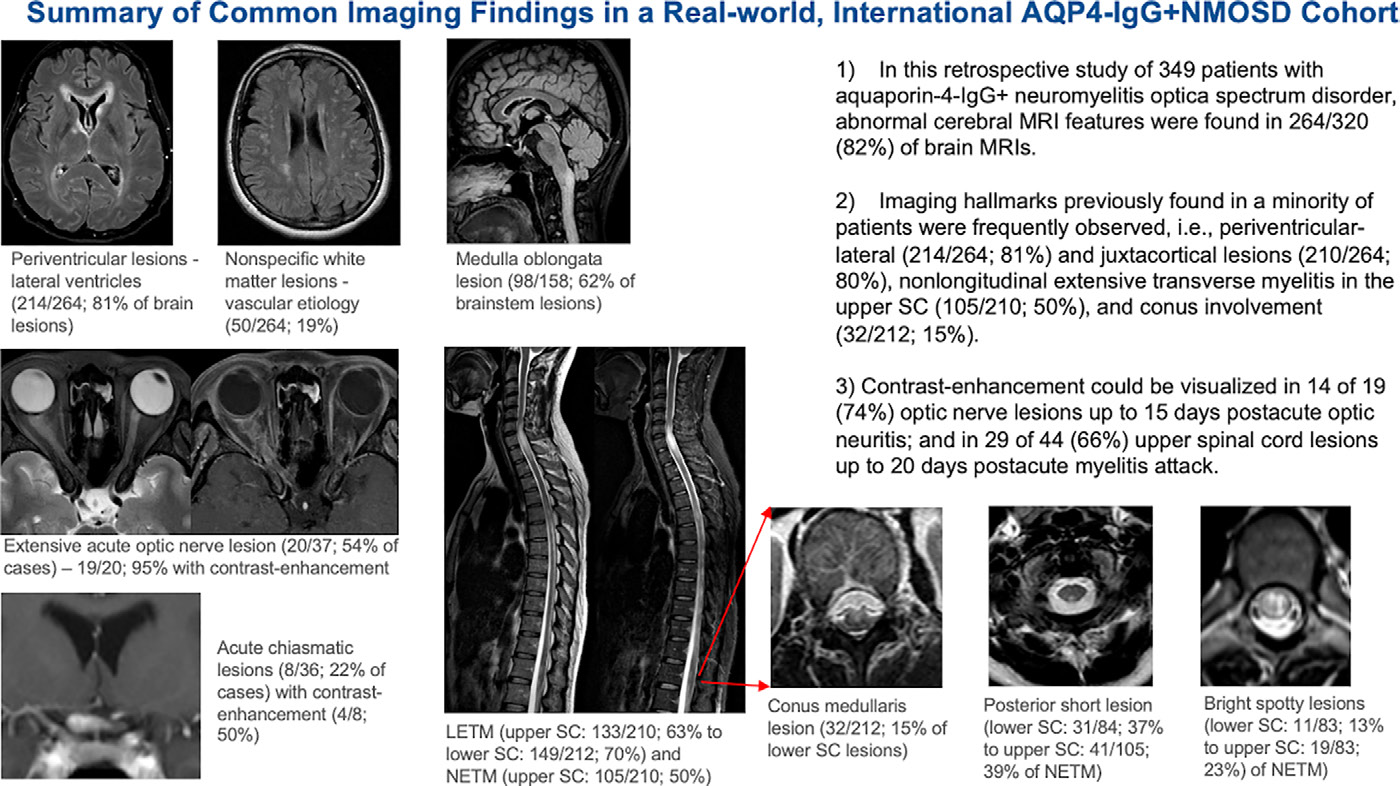
Summary of common imaging findings in a real-world, international cohort with aquaporin-4 immunoglobulin G–seropositive (AQP4-IgG+) neuromyelitis optica spectrum disorder (NMOSD). An enlarged image (arrows) shows the conus region of the spinal cord (SC). LETM = longitudinally extensive myelitis, NETM = nonlongitudinal extensive transverse myelitits.

Figure 6 illustrates a summary of common radiologic findings from this study.

## Discussion

We observed brain lesions to be the most frequent radiologic finding, differing from our meta-analysis results. Twenty percent of patients presented with imaging findings of small-vessel disease; spinal cord (SC) MRI showed longitudinally extensive transverse myelitis of the upper SC and/or lower SC, highly suggestive of neuromyelitis optica spectrum disorder. Nonlongitudinal extensive transverse myelitis was observed at a similar frequency.

Characteristic lesion morphologic structures, such as diencephalic lesions around the third ventricle and those affecting the area postrema (3), that were previously considered suggestive of NMOSD were considerably less frequent (eg, lateral ventricle–surrounding or subcortical lesions).

Typical imaging features of multiple sclerosis, such as the central vein sign or cortical lesions (7), were identified in one patient only. Generally, characteristics of multiple sclerosis such as central vein sign were barely observed in those patients with AQP4-IgG–seropositive NMOSD, corroborating previous studies (17). The relatively high frequency of white matter brain lesions resembling small-vessel disease in our cohort is important to note. We believe these findings represent the older age at onset of the population with NMOSD; however, we do not have enough information to understand how these observations differ from unspecific causes (eg, aging or migraine). Further exploration of possible vascular risk factors or pathologic mechanisms in NMOSD is therefore pertinent. We recommend higher-resolution fluid-attenuated inversion recovery and/or T2-weighted imaging sequences for accurate lesion depiction and to help with consistent longitudinal assessments.

Recently, Cortese et al (21) reported that deep gray matter atrophy may help to discriminate NMOSD from multiple sclerosis with moderate accuracy. For quantitative volumetric analyses, isotropic three-dimensional sequences are recommended and can be used to calculate lesion load, brain parenchymal gray and white matter volumes, or upper SC cross-sectional area (22). However, whether global or regional brain atrophy exists in patients with NMOSD (23) is debated; volumetric analyses may not be considered in clinical practice in the near future (24).

According to the multiple sclerosis–suggestive lesion criteria from Juryńczyk et al (25), the presence of simultaneous U fibers, Dawson finger–like lesions, at least one periventricular lesion, and one temporal lesion were observed in only five of 264 patients (1.9%). However, the periventricular changes exhibited a diffuse pattern with ependymal lining hyperintensity and wider lesion size or tumefactive lesions (26), which can be distinguishing features for patients with NMOSD who fulfill these criteria.

SC NETM lesions were often observed, and our data are in line with the clinical clues for NMOSD diagnosis proposed by Fang et al (14), who described more centrally located lesions in the SC. Therefore, our findings support the initial identification of short SC lesions as indications for NMOSD diagnosis (27).

Acute SC lesions persisted more frequently until 30 days after any attack (but especially after myelitis), although this was observed less frequently in the ON and/or brainstem. These observations are in line with those found in another cohort (28). Only two patients in the PAMRINO study with AQP4-IgG–seropositive NMOSD had available MRI to depict silent CE SC lesions (upper SC and lower SC). Another large study (29) found that 49% of patients with NMOSD had silent CE lesions during clinical attacks. Thus, full imaging coverage of the central nervous system is preferable (30).

Our study had several limitations. Considerable heterogeneity in the incidence of optic neuritis, myelitis, and abnormal MRI scans was found, likely because of both case ascertainment and clinical and/or MRI visit timing after a clinical attack. Also, because of the large time frame in which we matched MRI scans with data from corresponding clinical visits (within 180 days prior to or after the MRI examination), there is a chance that some acute MRI scans were mislabeled as chronic or nonacute. From our expert centers, we observed that patients are not always able to gain access to undergo MRI close to an attack that is not documented. Thus, we are confident that the misclassification of chronic scans or lesions as acute scans or lesions would be low. This finding is in line with another large study (31) in which attacks were documented and MRI was performed, but AQP4-IgG testing was the limiting factor required for correct diagnosis. In our study, the radiologist was also blinded to the radiologic readings used for the diagnosis. The MRI scans suggestive of acute lesions were analyzed, and lesions were confirmed as not silent or misread after unblinding the diagnoses. Therefore, through expert-center data collection, we were able to extract detailed clinical and MRI lesion information for profiling an orphan disease. Because few patients had new lesions and/or acute MRI scans, with mostly confirmed optic neuritis and/or myelitis attacks during the closest clinical visit, the dynamics of silent lesions on longitudinal MRI scans were not assessed. Other MRI studies found virtually no lesions (<3% of patients with AQP4-IgG–seropositive NMOSD), except few brain lesions, were clinically silent for longer than 3 months (30). More discussion regarding SC MRI and limitations at CE lesions and/or acute scans can be found in Appendix S1 (supplemental discussion and supplemental limitations sections).

The implementation of standardized imaging protocols and regular MRI follow-up in patients with neuromyelitis optica spectrum disorder is a future direction for disease monitoring (32). Large collaborative studies would benefit from standardized MRI protocols when planning for monitoring and volumetric central nervous system assessments, which will provide further insights into potential diffuse and/or subclinical disease activity (24).
